# Effectiveness of mulches in preventing *Popillia japonica* (Coleoptera: Scarabaeidae) oviposition in nursery potted plants

**DOI:** 10.1093/jee/toaf106

**Published:** 2025-06-13

**Authors:** Giovanni Dal Zotto, Thibault P M Costaz, Gabriele Pesavento, Klaas van Rozen, Herman H M Helsen, Paola Gotta, Beniamino Cavagna, Mariangela Ciampitti, Nicola Mori

**Affiliations:** Department of Biotechnology, University of Verona, Verona, Italy; Wageningen University and Research, Field Crops, Lelystad, The Netherlands; Agroscope, Route des Eterpys 18, Conthey, Switzerland; Department of Biotechnology, University of Verona, Verona, Italy; Wageningen University and Research, Field Crops, Lelystad, The Netherlands; Wageningen University and Research, Field Crops, Lelystad, The Netherlands; Piedmont Region, Plant Protection Service, Turin, Italy; Lombardy Region, Plant Protection Service, Milan, Italy; Lombardy Region, Plant Protection Service, Milan, Italy; DICATAM, University of Brescia, Brescia, Italy; Department of Biotechnology, University of Verona, Verona, Italy

**Keywords:** Japanese beetle, quarantine pest, pest-free trading, integrated pest management, physical barriers

## Abstract

*Popillia japonica* Newman (Coleoptera: Scarabaeidae) is a priority quarantine pest of the European Union that can pose an economic threat to certain agricultural activities. When female beetles have a choice, container substrates are not the preferred or suitable oviposition sites; however, plant nurseries are a potential pathway for spreading the pest. For this reason, producers must abide by restrictive measures to guarantee *P. japonica*-free plants, leading to major restrictions on their commercial activities. An innovative and sustainable approach to *P. japonica* control involves the application of a mulch layer to potted plants to physically prevent beetle oviposition. A choice test compared the effectiveness of 13 mulching materials in preventing *P. japonica* oviposition and the influence of soil moisture on egg laying. Additionally, for 10 of these materials, the role of mulch physical properties in preventing oviposition in potted plants was assessed in a no-choice test. The survey demonstrated that mulching can significantly reduce the risk of *P. japonica*’s oviposition in container substrates. Mulches with higher specific weights, such as gravel and lapilli pebbles, achieved greater control. Soil moisture influenced *P. japonica* oviposition, with higher moisture levels leading to increased egg laying. The application of suitable mulches represents an effective phytosanitary measure to improve the sustainable management of *P. japonica* in nurseries.

## Graphical Abstract

**Graphical Abstract:**
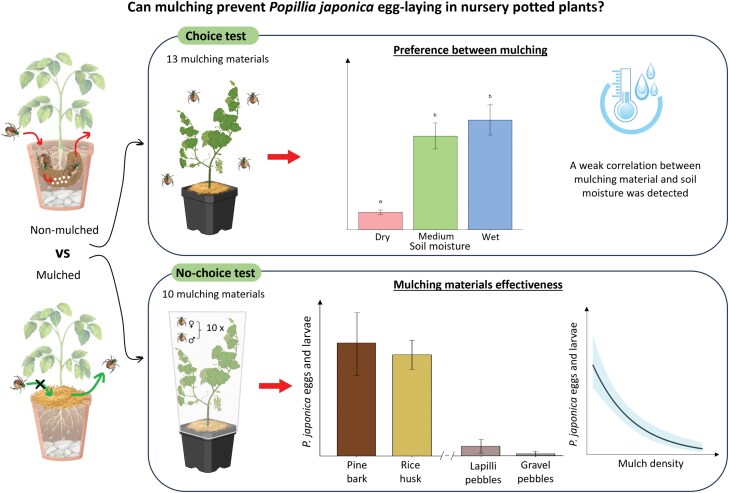

## Introduction

The Japanese beetle, *Popillia japonica* Newman (Coleoptera: Scarabaeidae), is an invasive polyphagous species native to Japan (Fleming 1972) that can damage more than 400 plant species, including crops, turf, ornamental and wild plants (Ladd 1987, 1989, Potter and Held 2002, Poggi et al. 2022). Since its accidental introduction in northern Italy in 2014 (EPPO 2014, Pavesi 2014), awareness of the threat *P. japonica* poses to European agriculture, landscape, and the economy has become more acute. The potential negative socio-economic impact of its spread led to its inclusion on the A2 list of priority quarantine organisms under delegated regulation (EU) 2023/1584 (EFSA PLH Panel 2018).

The local spread of *P. japonica* is facilitated by its ability to fly, allowing it to disperse up to 12 km per day (Lessio et al. 2022). Human activities, such as travel and trade, play a significant role in its transport over longer distances. *Popillia japonica* adults can exploit human mobility by hitchhiking rides on vehicles, while its eggs, larvae, and pupae can be dispersed by trading contaminated soil, potted, balled, and burlapped plants (EPPO Bulletin 2016, Poggi et al. 2022). In this context, the nursery plant production sector represents a particularly high-risk pathway for the spread of this quarantine pest (Mori et al. 2022, Gotta et al. 2023). Phytosanitary regulations (EU) 2023/1584 imposes severe limitations on plant producers located in areas of *P. japonica* outbreaks (EFSA PLH Panel 2018). While these measures mitigate the risk of pest dissemination, their application substantially increases costs for plant producers, as well as disruption to commercial activities, thereby compromising the stability of the entire European market in potted plants.

The regulation (EU) 2019/2072 mandates measures for producing *P. japonica*-free material, but their implementation is not always feasible. These regulations prohibit plant nurseries from selling plants outside the demarcated area unless they are sold as bare roots. Otherwise, producers must grow plants under complete insect-proof protection during the adult flight period to prevent *P. japonica* laying eggs in potted plants. The U.S. Domestic Japanese Beetle Harmonization Plan (DJBHP) recognizes broad-spectrum insecticide applications of soil media as the most effective strategy to ensure *P. japonica*-free potted plants (Mannion et al. 2001, Oliver et al. 2008, 2013, 2016, 2017). In Europe, however, the only soil treatments allowed in nursery production are based on entomopathogenic nematodes and fungi as plant protection products. Entomopathogenic nematodes, such as *Heterorhabditis bacteriophora* Poinar (Rhabditida: Heterorhabditidae) and *Steinernema carpocapsae* Weiser (Rhabditida: Steinernematidae) are effective in controlling *P. japonica* larvae in turfgrasses (Koppenhöfer and Fuzy 2004, Paoli et al. 2017, Marianelli et al. 2018, Torrini et al. 2020) but their use in nurseries requires further investigation due to the specific agronomic conditions. Entomopathogenic fungi, such as *Metarhizium* spp. (Hypocreales: Clavicipitaceae) and *Beauveria* spp. (Hypocreales: Cordycipitaceae) have demonstrated only limited effectiveness (Ramoutar et al. 2010, Giroux et al. 2015, Barzanti et al. 2023, Graf et al. 2023). The unavailability of effective plant protection products that can ensure the complete absence of *P. japonica* preimaginal stages in potted plants makes it necessary to explore alternative control strategies, such as physical methods.

Mulching can provide a protective layer between the soil and plants, acting as a physical barrier that interferes with the life cycles of subterranean insects (Muhammad et al. 2022). For example, Tartanus et al. (2017) investigated the efficacy of a fabric-like mulch in preventing egg-laying by *Melolontha* spp. (Coleoptera: Scarabaeidae) and the consequent presence of white grubs in the soil, observing a 2- to 3-fold increase in the number of larvae in non-mulched plots compared to mulched ones. In the case of *P. japonica*, Mori et al. (2022) tested coconut fiber discs, jute fabric and wood chips to inhibit oviposition by beetles inside netted potted plants. Encouraging results were obtained with coconut fiber discs which significantly reduced beetle oviposition. Only a limited number of materials were tested and the influence of their structural characteristics on the prevention of *P. japonica* egg laying in pots was overlooked.

Our research sought to further investigate the promising approach proposed by Mori et al. (2022) for preventing *P. japonica* oviposition on nursery plants, with a particular focus on the physical characteristics of mulches that may protect container substrates from infestation. We assessed the ability of *P. japonica* to lay eggs on those mulching materials widely used in nurseries and the influence of soil moisture on the egg laying by means of a choice test. Additionally, we evaluated the effects of the density, granulometry, and particle area of mulching material in a no-choice test in order to determine how such factors influence the success of *P. japonica* egg-laying in mulched potted plants. This study offers important insights into which physical properties should be considered when selecting the most effective mulch for producing potted plants free of *P. japonica* eggs and larvae.

## Materials and Methods

### Experimental Design and Setup

To assess the effectiveness of mulching in preventing *P. japonica* egg-laying in potted plants, a choice test and a no-choice test were performed. A total of 13 mulching materials commonly used in the nursery chain were evaluated (Table 1). Even though some studies demonstrated that plastic mulches could represent an effective physical barrier that can interfere with soil-dwelling insects (Bender et al. 2014, Prayogo et al. 2023), in this research, we preferred to evaluate eco-friendly materials. Indeed, plastic mulching is known to be a major source of macro and microplastic contamination in agroecosystems, with consequent negative effects on environmental health (Huang et al. 2020, Khalid et al. 2023).

**Table 1:** Characteristics of mulching materials in choice (C) and no-choice (NC) tests.

Mulching material	Trial	Manufacturer	Granulometry [mm]	Density [Kg/L]	Particle area [cm^2^]
Akadama	C—NC	Crespi Bonsai srl—Parabiago Milano—Italy	8 - 10	0.76	0.91
Beech chips	C—NC	AgriVivai srl—Pistoia—Italy	10 - 15	0.12	1.17
Gravel pebbles	C—NC	Granulati Zandobbio spa—Zandobbio Bergamo—Italy	7 - 15	1.39	2.09
Hemp chips	C—NC	Agritechnohouse srl—Carrara—Italy	10 - 15	0.10	1.28
Lapilli pebbles	C—NC	SEM Società estrattiva Monterosi srl—Monterosi Viterbo—Italy	7 - 10	0.82	0.62
Miscanthus chips	C—NC	AgriVivai srl—Pistoia—Italy	10 -15	0.12	0.68
Pine bark	C—NC	Terflor srl—Capriolo Brescia—Italy	5 - 50	0.23	3.14
Perlite	C—NC	*Agrilit3*—Perlite Italiana srl—Corsico Milan—Italy	2 - 6	0.07	0.09
Rice husk	C—NC	*Floriz* - Agromil Cereali srl—Gramellona Lomellina Pavia—Italy	1 - 2.5	0.11	0.15
Vermiculite	C—NC	*Vermex* - Soprema France—Strasbourg—France	0.5 - 3	0.16	0.05
Coconut fiber	C	Stocker Lana—Bolzano—Italy	Na	Na	Na
Biodegradable liquid mulch (BLM)	C	*Undisclosed information	Na	Na	Na
Pine wood chips	C	Van Egmond potgrond B.V. -Ankerweg Amsterdam -The Netherlands	15 -25	Na	Na

Values that are not applicable or not determined are marked as ‘Na’.

Both experiments were carried out in a nursery tunnel located in a *P. japonica* infestation area in the municipality of Bodio Lomnago (Varese, Italy; 45°46′59′′N 8°45′31′′ E). The 4- by 12-m tunnel was covered with an insect-proof net and a shadowing net on the roof. Access was only possible through a door and the tunnel floor was covered with a black plastic mulching film. Irrigation was provided by sprinklers on the ceiling to maintain plant turgidity. A data logger (RC-51H, Elitech, London, UK) was installed inside the tunnel to record environmental data (air temperature and relative humidity).

For both trials, 2-yr-old grape vine plants (Chardonnay grafted on SO4 rootstock) were grown in 11.7-liter black pots (diameter of top 26 cm). The plants were cultivated in a growing medium consisting of two-thirds field soil and one-third peat. The field soil was classified as silty clay loam (17.2% sand, 57.8% silt, and 25% clay), while the peat (Gramoflor GmbH & Co. KG, Vechta, Germany) was composed of 45% white peat, 30% black peat, 20% wood fiber, and 5% coconut fiber. To ensure that plants were free from *P. japonica* infestation, they were grown outside the infested area and transferred to the experimental site just before the trials began.

The different materials were applied in the pots to obtain a 5-cm thick mulching layer. Following the results obtained by Mori et al. (2022), coconut fiber discs (200 g/m², ~1 to 1.5-cm thick) were used as the reference mulching treatment and to enhance egg-laying prevention, excess flaps were secured to the pots with adhesive tape. A biodegradable liquid mulch (BLM) was also tested. This product, formulated to inhibit weed growth (composition not disclosed by the supplier), was applied to the soil surface of potted plants to create a layer measuring 1 to 2 cm. Potted plants with bare soil were considered as negative untreated control. Additionally, treatment with a cover of *Lolium* spp. was included by transplanting a grass sod into the pots. Potted plants were randomly placed inside the tunnel.

Healthy field-collected specimens of *P. japonica* were used for the two trials. On 19 July 2023, coinciding with the peak of the *P. japonica* flight, mating pairs of males and females of the beetle were collected from untreated wild vegetation near the nursery. The insects were allowed to feed and oviposit in the potted plants from 19 July to 3 August 2023 (*n* = 15 d). Thereafter, the efficacy of the different mulching materials in preventing *P. japonica* egg laying was assessed by counting the eggs and larvae in substrate beneath the mulching layer. A preliminary sampling of 10 pots revealed that most of the *P. japonica* eggs and larvae were concentrated in the upper 10 cm of substrate. Based on this observation, the top 15 cm of the substrate in each pot was inspected by four people simultaneously for at 10 min, ensuring a standardized assessment. To inspect the substrate, the upper 15 cm layer was sampled in all pots and since the number of eggs was very low, the entire soil of four pots per treatment was examined.

In the choice test, 10 *P. japonica* pairs (10 males and 10 females) were released inside the tunnel for each plant prepared for the trial. Each treatment was replicated eight times (ie eight pots), except for *Lolium* spp., which was replicated only four times to prevent its high attractiveness from influencing insect distribution. About the substrate inspection, considering the low number of eggs and larvae in this trial, in addition to the upper 15 cm section sampling, in four pots the entire soil volume was examined.

In the no-choice test, individual plants were enclosed in a net bag (70 by 100 cm, 1 mm mesh) to confine the beetles during the trial. 10 male and 10 female pairs were released into each caged potted plant for the no-choice test. All treatments were replicated four times except for *Lolium* spp., which was replicated eight times. Since BLM was an experimental product provided in limited quantities, only enough for the choice test, it was not possible to include it in this experiment. Regarding coconut fiber discs and pine wood chips, the former was not tested under no-choice conditions as it had already been evaluated under identical conditions in Mori et al. (2022), while the latter was excluded based on the assumption that its performance would be equivalent to that of pine bark.

### Soil Moisture Estimation

Considering the strong preference of *P. japonica* females for moist soil when selecting oviposition sites (Fleming 1972, Allsopp et al. 1992, Potter et al. 1996), an investigation was conducted on the role of soil moisture in *P. japonica* oviposition and whether it was correlated with the type of mulch used. This analysis was conducted in the choice test, where females were not forced to oviposit in the pot substrate and were free to select where to lay their eggs. The soil moisture was estimated using a visual soil assessment method, adapted from Shepherd et al. (2008), which was originally developed to assess soil suitability for ploughing. A small patch of substrate was rolled by hand into a cylindrical shape (a ‘worm’) to assess moisture content in each pot, classifying it as follows: *wet,* if the soil could be easily rolled into a continuous worm; *medium,* if the worm formed but broke into several sections; and *dry,* if the soil could not be shaped into a worm. Thereafter, using a soil humidity tester (YINMIK, Shandong Province, China), the substrate was classified as *dry* if it had a moisture level of less than 20%, *medium* between 20% and 35%, and *wet* more than 35%.

### Physical Properties of Mulching Material

To assess the role of the physical properties of mulching material in preventing *P. japonica* oviposition in the no-choice test, the analysis used the material density and the average granulometry values obtained from data provided by the manufacturers, as displayed on the product packaging or technical datasheets. When these were not displayed, the material density was calculated by weighing a known volume of the mulch. For the particle area, a 2D measurement of the particles was performed using the image analysis software ImageJ (Schneider et al. 2012). For each mulch, a perpendicular photo of the pot surface was taken to ensure the circular surface of the pot was not distorted in the photograph. We set the pot diameter (26 cm) as the scale reference in ImageJ and the perimeters of 30 randomly selected particles in each photo were traced to determine the average particle area.

### Statistical Analysis

The effectiveness of mulching materials in preventing *P. japonica* egg laying in container substrates was evaluated in both the choice and no-choice test using a generalized linear model (GLM) with a negative binomial distribution and a log error link to account for overdispersion in the data. The total number of eggs and larvae of *P. japonica* found in each pot was used as a response variable, while for the type of mulching material (factor with 15 and 13 levels, respectively for choice and no-choice tests), the pot inspection level (half or fully inspected) represented the explanatory variable in the choice test data analysis only.

Four separate GLMs with a negative binomial distribution and log link error were used to analyze the influence of soil moisture, material density, average granulometry, and particle area on beetle oviposition. The total number of eggs and larvae served as the response variable in all four models, while each of the aforementioned variables was used as explanatory variables. The correlation between soil moisture and mulching material was also checked using a Pearson’s Chi-squared test.

We verified the assumptions and validity of all models using the graphical display of the Pearson and scaled residuals. When categorical explanatory variables resulted in a significant effect (ie *P* < 0.05), a Tukey post hoc analysis with all pairwise comparisons was performed in order to gain further information on the effects of the various levels of the treatment. Given the high number of possible pairwise comparisons and the conservative nature of the adjustment, we chose to present Tukey-adjusted *P*-values. However, unadjusted *P*-values are also reported in the Supplementary Materials.

All the analyses were performed in R studio (version 4.3.1) (R Core Team 2021). To perform all the analyses, GLM with a negative binomial distribution was constructed using the function “glm.nb” from the “MASS” package (Ripley and Venables 2024). Pairwise comparisons were performed with the “emmeans” package (Lenth 2024). Models were checked for overdispersion and residual distribution using the “DHARMa” package (Hartig 2022). Graphics were produced using the ggplot2 package (Wickham 2024).

## Results

### Choice Test

**Fig. 1.**
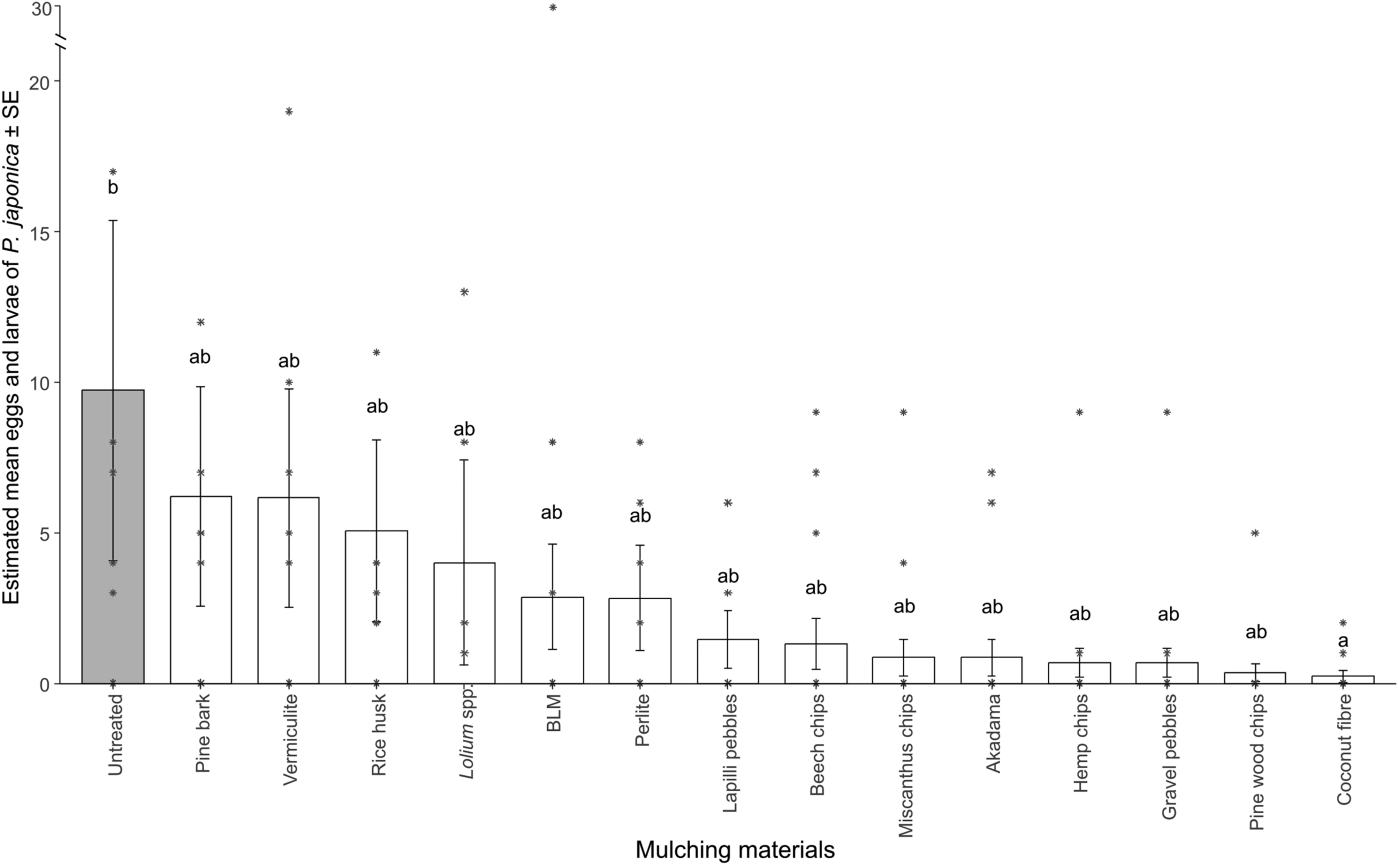
Estimated mean of eggs and larvae ± standard error laid by *Popillia japonica* in the soil of potted plants covered with different mulching materials in the choice test. Different letters indicate significant differences (*P* < 0.05) between mulching materials in pairwise comparison (Tukey *P*-value adjustment) from the negative binomial GLM. Asterisks represent observed data. The mulching material abbreviated as BLM corresponds to biodegradable liquid mulch.

In all, 327 eggs and larvae (106 and 222, respectively) were detected in the substrate beneath mulch of the potted plants used in the choice test. Biodegradable liquid mulch, *Lollium* spp., vermiculite, untreated, and pine bark allowed the highest egg deposition by *P. japonica*, showing a mean number of eggs and larvae ranging from 6.25 to 4.13. Rice husk, beech chips, perlite, and lapilli pebbles allowed the females to oviposit an average number of eggs in the container substrate of between 3.25 and 2.50. Plants mulched with akadama and miscanthus chips both had an average of 1.63 eggs and larvae, while hemp chips and gravel pebbles had 1.25. Plants mulched with pine wood chips and coconut fiber discs exhibited below the mulching layer only 0.63 and 0.38 eggs and larvae respectively (Supplementary Table S1). The estimated marginal means from the negative binomial GLM indicated that pots mulched with coconut fiber discs had statistically lower infestation by *P. japonica* than non-mulched ones. However, no differences were detected between all the other mulches (Fig. 1, Supplementary Table S2).

**Fig. 2.**
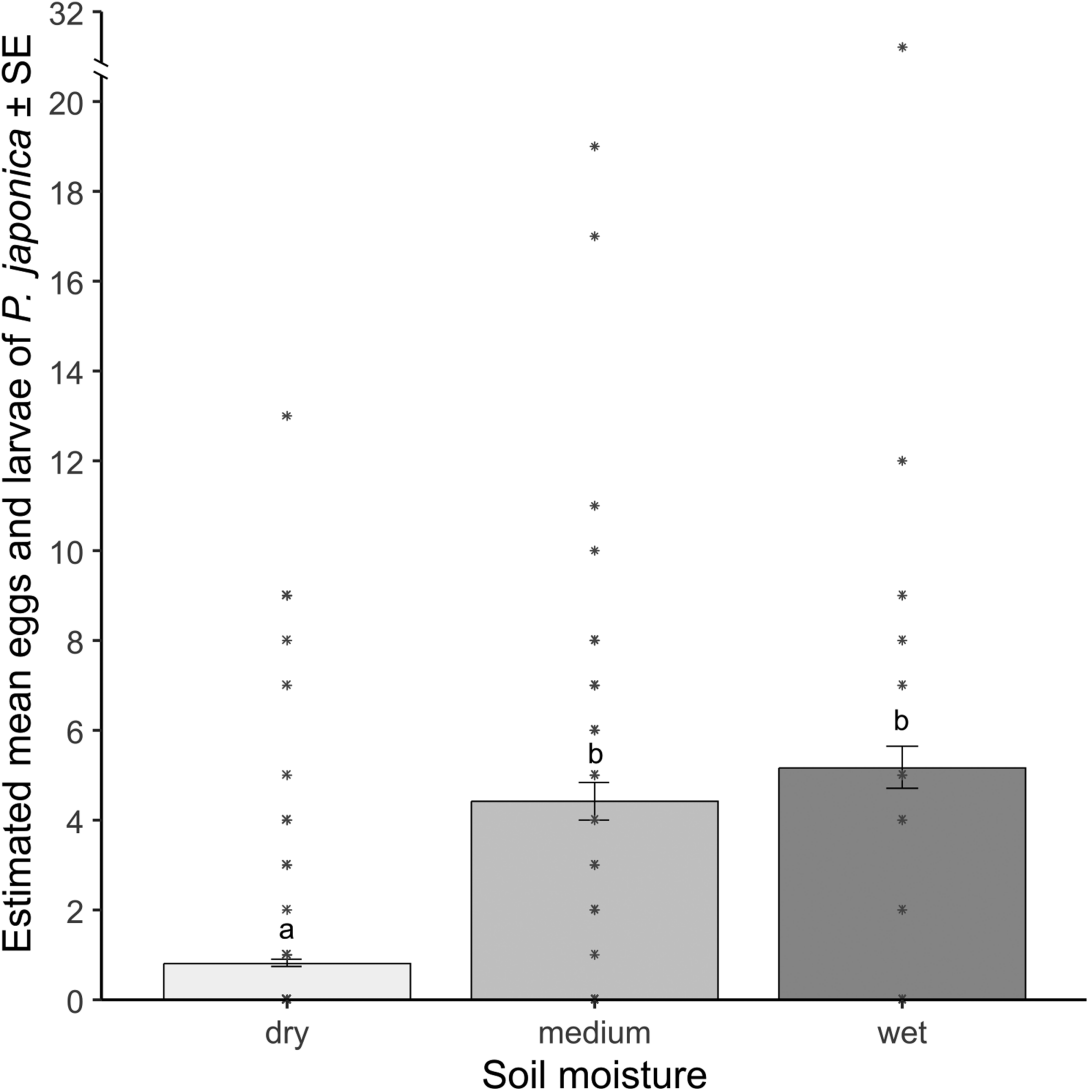
Estimated mean of eggs and larvae ± standard error laid by *Popillia japonica* in potted plants with different soil moisture levels. Different letters indicate statistical differences between soil moisture levels (*P* < 0.05) from the negative binomial GLM. Asterisks represent observed data.

The assessment of soil moisture in the potted plants revealed that *P. japonica* lay more eggs in pots with higher soil moisture levels. Females laid an average of 1.5 ± 0.4 eggs in 29.8% of pots categorized as dry, 3.8 ± 1.2 eggs in 60.9% of pots with medium moisture, and 4.8 ± 2.2 eggs in 55.5% of pots with wet soil (Fig. 2). The correlation of mulching material and pot humidity were near the predetermined 0.05 significance level (χ² = 40, df = 28, *P* = 0.07).

### No-choice Test

**Fig. 3.**
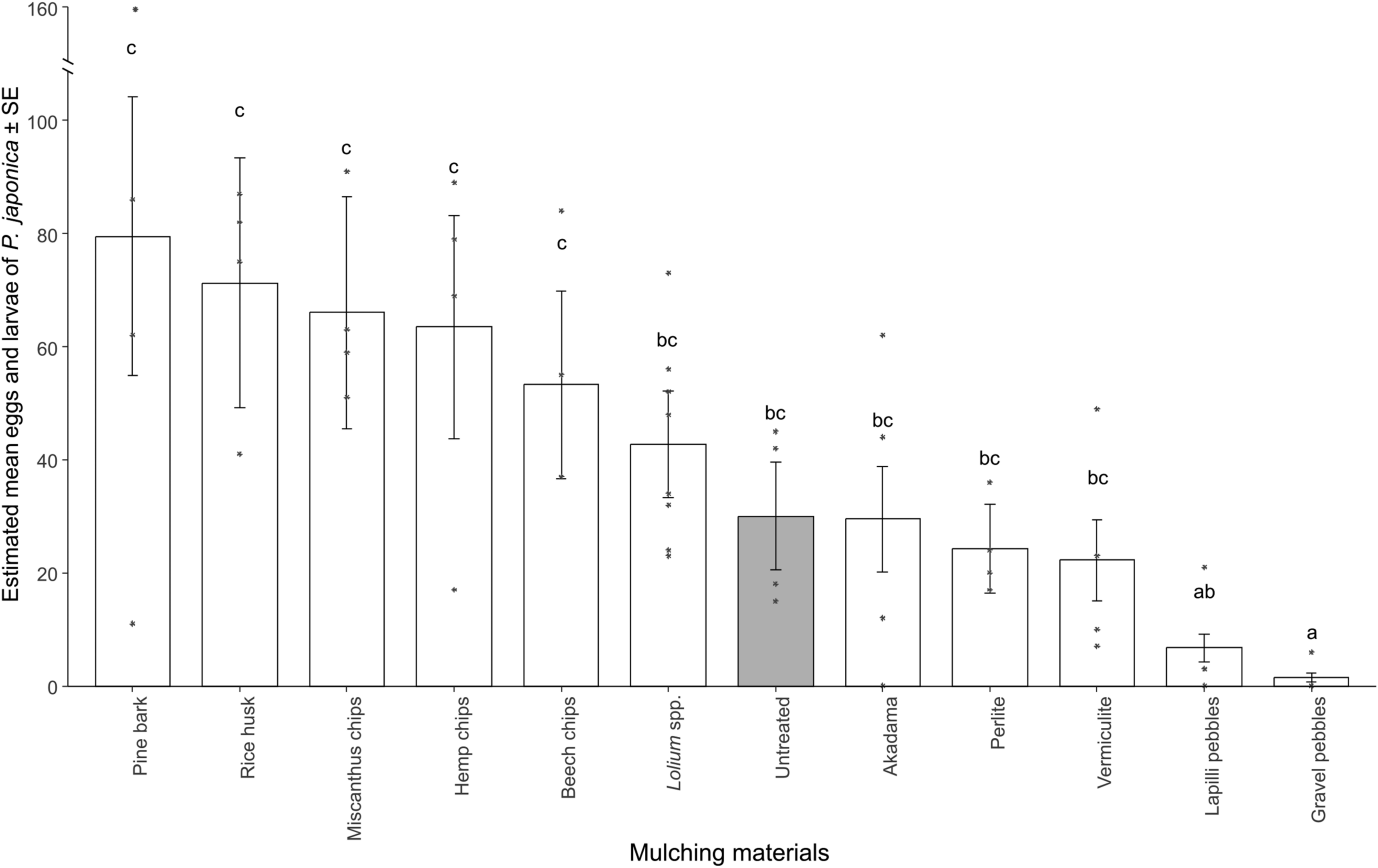
Estimated mean of eggs and larvae ± standard error laid by *Popillia japonica* in the soil of potted plants covered with different mulching materials in the no-choice test. Different letters indicate significant differences (*P* < 0.05) between mulching materials in pairwise comparison (Tukey *P*-value adjustment) from the negative binomial GLM. Asterisks represent observed data. The mulching material abbreviated as BLM corresponds to biodegradable liquid mulch.

In all, 2,133 *P. japonica* eggs and larvae (1,696 and 437, respectively) were counted. Soil of potted plants mulched with pine bark, rice husk, miscanthus chips, hemp chips, and beech chips displayed an average number of eggs and larvae ranging from 79.50 to 53.25. *Lolium* spp., untreated, akadama, perlite, and vermiculite yielded between 42.75 to 22.25 beetle eggs and larvae in the soil beneath the mulch while lapilli and gravel pebbles recorded only 6.75 and 1.50, respectively (Supplementary Table S3). All mulching treatments, except for akadama, lapilli, and gravel pebbles, displayed 100% of pots in which soil resulted infested by *P. japoni*ca. Both akadama and lapilli pebbles produced 75% infested pots (3 out of 4), while gravel pebbles had 25% (1 out of 4) infested pots. The estimated marginal means from the negative binomial GLM demonstrated that gravel pebbles are the most effective mulching material in reducing *P. japonica* oviposition, while lapilli pebbles were shown to be statistically more effective than pine bark, rice husk, miscanthus chips, hemp chips, and beech chips (Fig. 3, Supplementary Table S4).

**Fig. 4.**
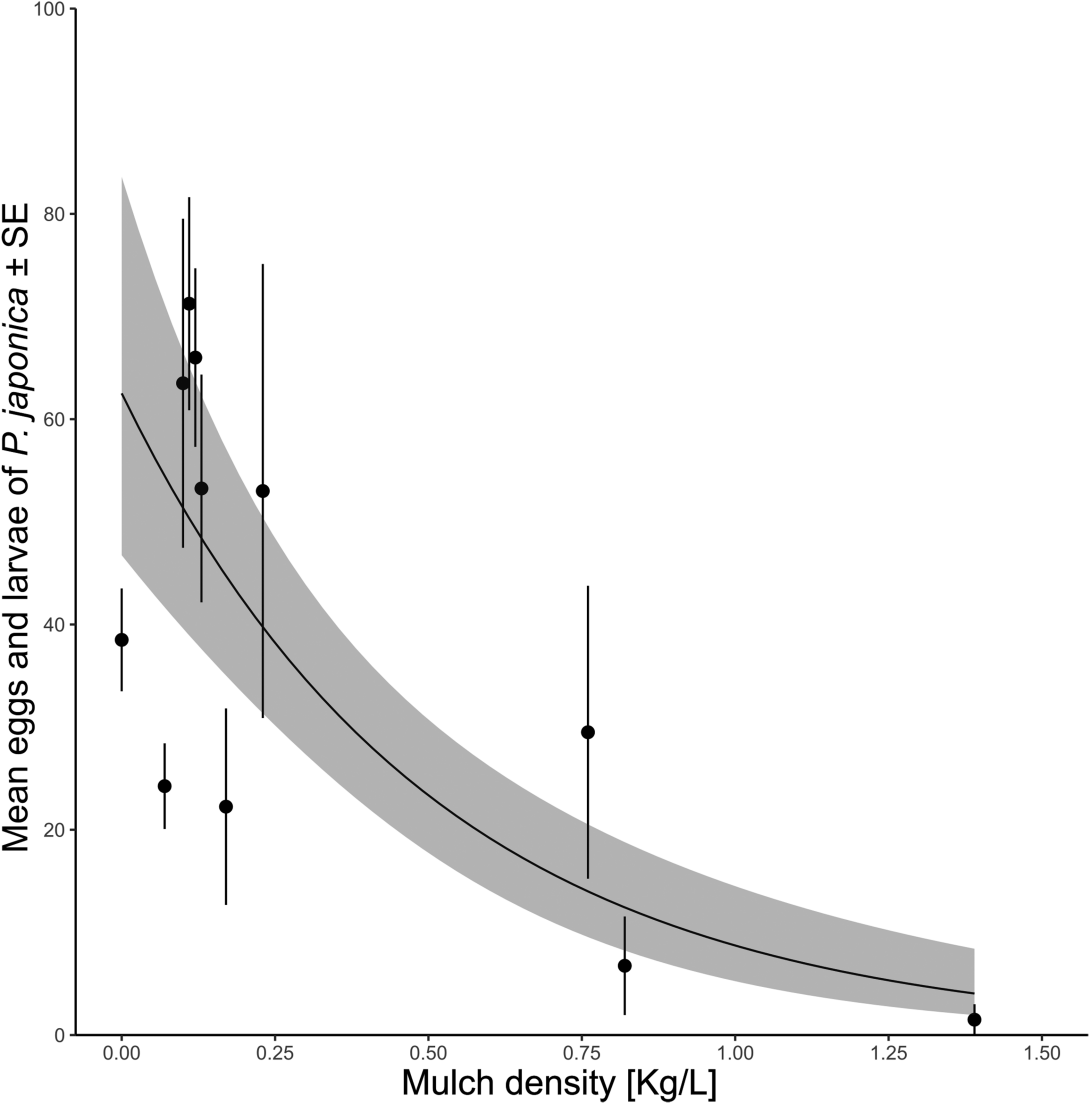
Effect of mulching material density on egg-laying of *Popillia japonica* in mulched potted plants in the no-choice test. Dots and bars represent the average numbers of *P. japonica* eggs and larvae, with the standard error around the mean. The line is the regression curve estimated by the model, and the shaded area represents the 95% confidence intervals of the regression model.

The analysis of the physical properties of mulch revealed that the material density can affect the oviposition success of *P. japonica* in potted plants (*P* < 0.001). Indeed, higher specific weight led to fewer beetle eggs and larvae in pots (Fig. 4). On the other hand, no correlation was detected between *P. japonica* egg laying and mulching material with average granulometry (*P* = 0.22) and particle area (*P* = 0.41) (Supplementary Figs. S1 and S2).

## Discussion

*Popillia japonica* is likely to have a remarkable impact on the Italian and European nursery industries. As this quarantine pest continues to expand its geographical range (EPPO, 2022, 2023, Gotta et al. 2023), it represents an increasing and spreading threat to the nursery plant production sector, making the development of new management strategies a matter of urgency. This study performed a comprehensive screening of a wide range of natural mulching materials with the potential to prevent *P. japonica* egg deposition in potted plant substrate and offers valuable recommendations for the nursery industry. Although none of the tested materials fully prevented *P. japonica* egg deposition, mulching had a significant impact in reducing egg deposition in the substrate of potted plants. In the choice test, coconut fiber discs, pine wood chips, and gravel pebbles were the materials that led to the lowest *P. japonica* substrate infestation, while in the no-choice test, gravel and lapilli pebbles were the most effective materials in preventing pot infestation, exhibiting the lowest number of eggs and larvae. Further analysis of the cover materials regarding soil moisture, average granulometry, particle area, and material density revealed that soil moisture affected the number of eggs and larvae in potted plants, with a slight correlation observed between the type of mulching material and the moisture levels. Mulch density had a significant negative effect on the number of eggs and larvae, with denser mulches resulting in fewer *P. japonica* eggs and larvae.

The choice test confirmed the well-known tendency of *P. japonica* females to search for short, grass-covered areas to lay their eggs (Potter and Held 2002, Szendrei and Isaacs 2005, Wood et al. 2009); indeed, pots containing *Lolium* spp. grass contained a high number of eggs. This can be explained by the *optimal oviposition* (Jaenike, 1978) or the *mother-knows-best* theories (Valladares and Lawton 1991), both of which suggest that females choose oviposition sites that ensure the maximum survival rate and fitness of their offspring. These principles are highly relevant in the case of insects whose larvae have limited mobility (Clark et al. 2011), such as *P. japoni*ca. Additionally, Szendrei and Isaacs (2005) hypothesized that vertical objects in the soil surface, such as grass, serve as visual cues that guide *P. japonica* in selecting egg-laying sites and digging behavior.

Soil moisture can be a physical cue at the ground level and is a good candidate to explain oviposition site selection by females seeking the best chances of survival of their offspring. In our work, more *P. japonica* eggs and larvae were recovered in pots with moderate to high soil moisture levels, confirming previous studies (Fleming 1972, Allsopp et al. 1992, Potter et al. 1996). In the case of the Japanese beetle, moist soil may be preferred as a way of reducing the desiccation and consequent mortality of eggs and larvae. Indeed, low larval densities are detected in drought soil (Allsopp et al. 1992, Kistner-Thomas, 2019, Simonetto et al. 2022).

Even though our analysis revealed only a weak correlation between mulching materials and soil moisture, the infestation of potted plant substrate by *P. japonica* may be driven by the level of soil moisture provided by the mulching material. Fewer eggs and larvae were found in the soil of the pots mulched with coconut fiber discs, pine wood chips, gravel pebbles, and hemp chips, which displayed the most pots with dry soil. In contrast, vermiculite, pine bark, and rice husk, which produced a majority of wet and medium moisture pots (Supplementary Table S5), exhibited the highest numbers of *P. japonica* eggs and larvae. Although we did not directly measure the moisture of the mulches, it is reasonable to assume that it was correlated with that of the underlying soil. For example, rock-fragment mulches which have low water retention, provided lower soil moisture compared with organic mulches (Jiménez et al. 2017). Therefore, since the mulch is the first substrate encountered by *P. japonica* females, its moisture perception may act as a cue for initiating or avoiding the oviposition. This is supported by studies conducted on other beetles showing that organic mulches with high moisture promote infestations of the banana weevil, *Cosmopolites sordidus* Germar (Coleoptera: Curculionidae) through enhancing the moisture of underlying substrate (Gold et al. 2006).

The mulches affected *P. japonica* oviposition ability acting as a physical barrier or influencing the selection of egg-laying sites. Additionally, the effectiveness of some mulching materials may be linked to their role in increasing post-ovipositional mortality. Although no dead eggs and larvae were detected, it is well known that mulching causes microclimatic modifications that may create either favorable or adverse conditions for the development and survival of soilborne pests (Rendon et al. 2020). Covering the soil surface with mulching materials can prevent rapid temperature fluctuations, direct sunlight, and desiccation (de Waal et al. 2011), which may benefit soilborne pests. Conversely, the mulching leads to increased soil temperatures, becoming lethal to the young stages. Therefore, to effectively control *P. japonica* oviposition in potted plants through mulching, it is essential to use materials that modify the soil environment in ways that discourage egg laying or that can favor post-ovipositional mortality.

Beyond acting as a physical barrier and creating unfavorable conditions for soilborne pests, the volatile profile of plant-based mulches may also influence *P. japonica* behavior, either attracting or repelling it. However, our experimental setup, in which plants were placed close to each other, did not allow us to assess this aspect. Nevertheless, studies on *P. japonica* chemical ecology showed that the Japanese beetle attraction to organic volatiles is primarily associated with compounds emitted by herbivore-damaged plants, rather than those resulting from mechanical damage (Loughrin et al. 1995, Noge et al. 2011).

The no-choice test provided clear information regarding the effectiveness of the tested mulching materials in inhibiting the egg deposition of *P. japonica* in potted plants. The mulch effectiveness was related to the physical characteristics of the materials; pine bark, rice husk, and wood chips (miscanthus, hemp, and beech) performed less well than rocky materials, especially gravel. *Popillia japonica* egg-laying reduction obtained by lapilli (6.75 ± 4.8 eggs) and gravel pebbles (1.50 ± 1.5 eggs) was consistent with the best results achieved by Mori et al. (2022) with 200 g/m^2^ (5.5 ± 3.3 larvae) and 400 g/m^2^ (1.75 ± 1.4 larvae) coconut fiber discs. Moreover, gravel was the only material that displayed only 25% of infestation.

The particle area and average granulometry of mulch had no impact on the oviposition of *P. japonica*, but the density of the mulching material did play a significant role in reducing the risk of pot contamination. The most effective mulching materials, gravel, and lapilli pebbles, had the highest specific weights at 1.39 kg/L and 0.82 kg/L, respectively. Heavier particles are more difficult for the beetles to move and so could limit their access to the soil. In contrast, lighter materials, such as rice husks, miscanthus, hemp, and beech chips, provided minimal substrate protection in the no-choice test. In the case of pine bark mulching, the empty spaces between adjacent particles may have facilitated beetle penetration and subsequent oviposition, which could explain the poor performance of this material in both experiments. Similar observations have been reported for *Dasylepida ishigakiensis* Niijima and Kinoshita (Coleoptera: Scarabaeidae) adults, which demonstrated enhanced digging ability in coarse-grained substrates compared to fine-grained ones (Harano et al. 2010).

Overall, the incomplete oviposition prevention achieved by the mulching materials tested is due to the extremely high densities of *P. japonica* confined within the tunnel and in the caged plants. Such a high and prolonged level of infestation would be unlikely to persist within a nursery greenhouse during the adult flight period due to insecticide applications and agronomic practices that keep the pest population density low. Moreover, this study was conducted using a plant species highly attractive to *P. japonica* as a food source, which may have enhanced the insect’s fitness and subsequently increased oviposition rates. Since this research is based on a worst-case scenario for the nursery chain in a *P. japonica* outbreak area, it provides useful information for decision-makers in order to refine measures for growing and trading non-infested plants.

Even though some tested mulching materials showed promising results in limiting *P. japonica* egg laying in potted plants, practical considerations must be taken into account when used within the nursery chain. Adoption of gravel or lapilli as mulches could significantly increase the amount of effort required to handle potted plants. Other factors also come into play, such as the cost of the mulching material, the labor involved in their application, the economic value of the plant to be protected, but also the positive effects brought by mulch (Barche et al. 2015). Additionally, certain ornamental plants may also present a higher risk of infestation and should be subject to regulatory measures to minimize pest spread. For instance, under the U.S. DJBHP, potted plants from the Poaceae and Cyperaceae families are not certified as *P. japonica*-free, even when grown in approved substrates and under certified production protocols, due to their high suitability for larval development.

The findings of this study can provide valuable insights for improving the sustainable management of *P. japonica* in the nursery industry, as well as confirming the validity of mulching as a phytosanitary risk management measure within a system approach. With regard to natural infestation, the adequate control of weeds in potted plants, appropriate irrigation management, and the selection of suitable mulching materials could significantly reduce the risk of oviposition in pots. Since the adult stage of *P. japonica* is associated with high temperatures, further studies are required to evaluate appropriate irrigation management techniques that can limit *P. japonica* oviposition without compromising plant health. Additional research is required to determine the optimal thickness of mulch that can effectively prevent *P. japonica* oviposition and to explore the combination of this technique with the use of entomopathogenic nematodes or fungi.

## Supplementary material

Supplementary material is available at *Journal of Economic Entomology* online.

toaf106_suppl_Supplementary_Material

## References

[CIT0001] Allsopp PG, Klein MG, McCoy EL. 1992. Effect of soil moisture and soil texture on oviposition by Japanese beetle and Rose chafer (Coleoptera: Scarabaeidae). J. Econ. Entomol. 85:2194–2200. https://doi.org/10.1093/jee/85.6.2194

[CIT0002] Barche S, Nair R, Jain PK. 2015. A review of mulching on vegetable crops production. Eco Env Cons. 21:859–866. https://doi.org/10.13140/RG.2.2.14223.33440

[CIT0003] Barzanti GP, Enkerli J, Benvenuti C, et al 2023. Genetic variability of *Metarhizium* isolates from the Ticino Valley Natural Park (Northern Italy) as a possible microbiological resource for the management of *Popillia japonica*. J. Invertebr. Pathol. 197:107891. https://doi.org/10.1016/j.jip.2023.107891 36716929

[CIT0004] Bender GS, Bates LM, Bethke JA, et al 2014. Evaluation of insecticides, entomopathogenic nematodes, and physical soil barriers for control of *Diaprepes abbreviatus* (Coleoptera: Curculionidae) in citrus. J. Econ. Entomol. 107:2137–2146. https://doi.org/10.1603/EC14150 26470079

[CIT0005] Clark KE, Hartley SE, Johnson SN. 2011. Does mother know best? The preference–performance hypothesis and parent–offspring conflict in aboveground–belowground herbivore life cycles. Ecol. Entomol. 36:117–124. https://doi.org/10.1111/j.1365-2311.2010.01248.x

[CIT0057] de Waal JY, Malan AP, Addison MF. 2011. Evaluating mulches together *with Heterorhabditis zealandica* (Rhabditida: Heterorhabditidae) for the control of diapausing codling moth larvae, *Cydia pomonella* (L.) (Lepidoptera: Tortricidae). Biocontrol Sci. Technol. 21:255–270. https://doi.org/10.1080/09583157.2010.540749

[CIT0006] EFSA PLH Panel. 2018. Pest categorisation of *Popillia japonica*. EFSA J. 16:e05438. https://doi.org/10.2903/j.efsa.2018.5438 32625740 PMC7009705

[CIT0007] EPPO. 2014. First report of *Popillia japonica* in Italy. EPPO Report. Serv. no. 10-2014 Article num: 2014/179. Available from https://gd.eppo.int/reporting/article-3272

[CIT0009] EPPO. 2022. Update of the situation of *Popillia japonica* in Italy. EPPO Report. Serv. no. 04-2022 Article num: 2022/081. Available from https://gd.eppo.int/reporting/article-7312

[CIT0010] EPPO. 2023. Update of the situation of *Popillia japonica* in Switzerland. EPPO Report. Serv. no. 08-2023 Article num: 2023/184. Available from https://gd.eppo.int/reporting/article-7666

[CIT0008] EPPO Bulletin. 2016. PM 9/21(1) *Popillia japonica*: procedures for official control. EPPO Bull. 46:543–555. https://doi.org/10.1111/epp.12345

[CIT0011] EU. 2019. Commission Implementing Regulation (EU) 2019/2072 of 28 November 2019 establishing uniform conditions for the implementation of Regulation (EU) 2016/2031 of the European Parliament and the Council, as regards protective measures against pests of plants, and repealing Commission Regulation (EC) No 690/2008 and amending Commission Implementing Regulation (EU) 2018/2019. OJ L 319:1–279. http://data.europa.eu/eli/reg_impl/2019/2072/oj

[CIT0012] EU. 2023. Commission Implementing Regulation (EU) 2023/1584 of 1 August 2023 on measures to prevent the establishment and spread of *Popillia japonica* Newman and on measures for the eradication and containment of that pest within certain demarcated areas of the Union territory. OJL 194:17–38. http://data.europa.eu/eli/reg_impl/2023/1584/oj

[CIT0013] Fleming WE. 1972. Biology of the Japanese beetle. US Department of Agriculture - ARS Technical Bulletin No. 1449. US GPO.

[CIT0014] Giroux F, Lavallée R, Bauce E, et al 2015. Susceptibility of the Japanese beetle, *Popillia japonica* (Newman) (Coleoptera: Scarabaeidae), to entomopathogenic Hypocreales fungi. Phytoprotection 95:1–6. https://doi.org/10.7202/1028399ar

[CIT0015] Gold CS, Okech SH, McIntyre BD, et al 2006. Effects of mulch on banana weevil *Cosmopolites sordidus* (Germar) populations and damage in Uganda. Crop Prot. 25:1153–1160. https://doi.org/10.1016/j.cropro.2005.10.013

[CIT0016] Gotta P, Ciampitti M, Cavagna B, et al 2023. *Popillia japonica* – Italian outbreak management. Front. Insect Sci 3:1175138. https://doi.org/10.3389/finsc.2023.1175138. Available from https://www.frontiersin.org/articles/10.3389/finsc.2023.1175138 38469512 PMC10926379

[CIT0017] Graf T, Scheibler F, Niklaus PA, et al 2023. From lab to field: biological control of the Japanese beetle with entomopathogenic fungi. Front. Front. Insect Sci. 3:1138427. https://doi.org/10.3389/finsc.2023.1138427 38469508 PMC10926434

[CIT0018] Harano KI, Tanaka S, Tokuda M, et al 2010. Factors influencing adult emergence from soil and the vertical distribution of burrowing scarab beetles *Dasylepida ishigakiensis*. Physiol. Entomol. 35:287–295. https://doi.org/10.1111/j.1365-3032.2010.00741.x

[CIT0019] Hartig F. 2022. DHARMa: Residual diagnostics for hierarchical (Multi-Level / Mixed) regression models.:0.4.6. https://doi.org/10.32614/CRAN.package.DHARMa

[CIT0020] Huang Y, Liu Q, Jia W, et al 2020. Agricultural plastic mulching as a source of microplastics in the terrestrial environment. Environ. Pollut. 260:114096. https://doi.org/10.1016/j.envpol.2020.114096 32041035

[CIT0021] Jaenike J. 1978. On optimal oviposition behavior in phytophagous insects. Theor. Popul. Biol. 14:350–356. https://doi.org/10.1016/0040-5809(78)90012-6 751265

[CIT0022] Jiménez MN, Pinto JR, Ripoll MA, et al 2017. Impact of straw and rock-fragment mulches on soil moisture and early growth of holm oaks in a semiarid area. CATENA 152:198–206. https://doi.org/10.1016/j.catena.2017.01.021

[CIT0023] Khalid N, Aqeel M, Noman A, et al 2023. Impact of plastic mulching as a major source of microplastics in agroecosystems. J. Hazard. Mater. 445:130455. https://doi.org/10.1016/j.jhazmat.2022.130455 36463747

[CIT0024] Kistner-Thomas EJ. 2019. The potential global distribution and voltinism of the Japanese beetle (Coleoptera: Scarabaeidae) under current and future climates. J. Insect Sci. 19:16. https://doi.org/10.1093/jisesa/iez023 PMC6429693 30900722

[CIT0025] Koppenhöfer AM, Fuzy EM. 2004. Effect of white grub developmental stage on susceptibility to entomopathogenic nematodes. J. Econ. Entomol. 97:1842–1849. https://doi.org/10.1603/0022-0493-97.6.1842 15666735

[CIT0026] Ladd TL. 1987. Japanese beetle (Coleoptera: Scarabaeidae): influence of favored food plants on feeding response. J. Econ. Entomol. 80:1014–1017. https://doi.org/10.1093/jee/80.5.1014

[CIT0027] Ladd TL. 1989. Japanese beetle (Coleoptera: Scarabaeidae): feeding by adults on minor host and nonhost plants. J. Econ. Entomol. 82:1616–1619. https://doi.org/10.1093/jee/82.6.1616

[CIT0028] Lenth RV. 2024. Emmeans: estimated marginal means, aka least-squares means. R package version: 1.10.5. https://rvlenth.github.io/emmeans/

[CIT0029] Lessio F, Pisa CG, Picciau L, et al 2022. Feb 11. An immunomarking method to investigate the flight distance of the Japanese beetle. Entomol. Gen. 42:45–56. https://doi.org/10.1127/entomologia/2021/1117

[CIT0030] Loughrin JH, Potter DA, Hamilton-Kemp TR. 1995. Volatile compounds induced by herbivory act as aggregation kairomones for the Japanese beetle (*Popillia japonica* Newman). J. Chem. Ecol. 21:1457–1467. https://doi.org/10.1007/BF02035145 24233676

[CIT0031] Mannion CM, McLane W, Klein MG, et al 2001. Management of early-instar Japanese beetle (Coleoptera: Scarabaeidae) in field-grown nursery crops. J. Econ. Entomol. 94:1151–1161. https://doi.org/10.1603/0022-0493-94.5.1151 11681678

[CIT0032] Marianelli L, Paoli F, Torrini G, et al 2018. Entomopathogenic nematodes as potential biological control agents of *Popillia japonica* (Coleoptera, Scarabaeidae) in Piedmont Region (Italy). J. Appl. Entomol. 142:311–318. https://doi.org/10.1111/jen.12470

[CIT0033] Mori N, Santoiemma G, Glazer I, et al 2022. Management of *Popillia japonica* in container-grown nursery stock in Italy. Phytoparasitica 50:83–89. https://doi.org/10.1007/s12600-021-00948-2

[CIT0034] Muhammad A, Ali M, Shakeel M, et al 2022. Comparative effects of living and non-living mulches on insect pest management in agroecosystems. In: Akhtar K, Arif M, Riaz M, et al, editors. Mulching in Agroecosystems. Springer Nature Singapore. p. 231–248. https://doi.org/10.1007/978-981-19-6410-7_15

[CIT0035] Noge K, Abe M, Tamogami S. 2011. Phenylacetonitrile from the Giant Knotweed, *Fallopia sachalinensis*, infested by the Japanese Beetle, *Popillia japonica*, is induced by exogenous methyl hasmonate. Molecules 16:6481–6488. https://doi.org/10.3390/molecules16086481 21814160 PMC6264294

[CIT0036] Oliver JB, Reding ME, Dennis SO, et al 2008. Drench treatments for management of larval Japanese beetle (Coleoptera: Scarabaeidae) in field-grown balled and burlapped nursery plants. J. Econ. Entomol. 101:1158–1166. https://doi.org/10.1603/0022-0493(2008)101[1158:dtfmol]2.0.co;2. 18767724

[CIT0037] Oliver JB, Ranger CM, Reding ME, et al 2013. Preharvest quarantine treatments of chlorantraniliprole, clothianidin, and imidacloprid-based insecticides for control of Japanese beetle (Coleoptera: Scarabaeidae) and other scarab larvae in the root zone of field-grown nursery trees. J. Econ. Entomol. 106:1190–1199. https://doi.org/10.1603/EC13059. 23865183

[CIT0038] Oliver JB, Reding ME, Ranger CM, et al 2016. Insecticides evaluated as regulatory immersion treatments to eliminate third-instar Japanese beetles (Coleoptera: Scarabaeidae) from small-diameter field-grown nursery plants. J. Entomol. Sci. 51:9–28. https://doi.org/10.18474/jes15-13.1.

[CIT0039] Oliver JB, Ranger CM, Reding ME, et al 2017. Insecticides and their combinations evaluated as regulatory immersion treatments for third-instar Japanese beetle (Coleoptera: Scarabaeidae) in field-grown and containerized nursery plants. J. Entomol. Sci. 52:274–287. https://doi.org/10.18474/jes17-18.1.

[CIT0040] Paoli F, Marianelli L, Torrini G, et al 2017. Differential susceptibility of *Popillia japonica* 3rd instars to *Heterorhabditis bacteriophora* (Italian strain) at three different seasons. Biocontrol Sci. Technol. 27:439–444. https://doi.org/10.1080/09583157.2017.1285866.

[CIT0041] Pavesi M. 2014. *Popillia japonica*, specie aliena invasiva segnalata in Lombardia. Inf. Agrar. 32:53–55.

[CIT0042] Poggi S, Desneux N, Jactel H, et al 2022. A nationwide pest risk analysis in the context of the ongoing Japanese beetle invasion in Continental Europe: The case of metropolitan France. Front. Front. Insect Sci. 2:1079756. https://doi.org/10.3389/finsc.2022.1079756 38468800 PMC10926453

[CIT0043] Potter DA, Held DW. 2002. Biology and management of the Japanese beetle. Annu. Rev. Entomol. 47:175–205. https://doi.org/10.1146/annurev.ento.47.091201.145153 11729073

[CIT0044] Potter DA, Powell AJ, Spicer PG, et al 1996. Cultural practices affect root-feeding white grubs (Coleoptera: Scarabaeidae) in turfgrass. J. Econ. Entomol. 89:156–164. https://doi.org/10.1093/jee/89.1.156

[CIT0045] Prayogo Y, Bayu MSYI, Indiati SW. 2023. Control measure of sweet potato weevil (*Cylas formicarius* Fab.) (Coleoptera: Curculionidae) in endemic land of entisol type using mulch and entomopathogenic fungus *Beauveria bassiana*. Open Agric. 8(1):20220237. https://doi.org/10.1515/opag-2022-0237

[CIT0046] R Core Team. 2021. R: a language and environment for statistical computing. Available from https://www.r-project.org/

[CIT0047] Ramoutar D, Legrand AI, Alm SR. 2010. Field performance of *Metarhizium anisopliae* against *Popillia japonica* (Coleoptera: Scarabaeidae) and *Listronotus maculicollis* (Coleoptera: Curculionidae) larvae in turfgrass. J. Entomol. Sci. 45:20–26. https://doi.org/10.18474/0749-8004-45.1.20

[CIT0048] Rendon D, Hamby KA, Arsenault-Benoit AL, et al 2020. Mulching as a cultural control strategy for *Drosophila suzukii* in blueberry. Pest Manag. Sci. 76(1):55–66. https://doi.org/10.1002/ps.5512. 31207075

[CIT0049] Ripley B, Venables B. 2024. MASS: Support functions and datasets for Venables and Ripley’s MASS.:7.3–761. https://doi.org/10.32614/CRAN.package.MASS

[CIT0050] Schneider CA, Rasband WS, Eliceiri KW. 2012. NIH Image to ImageJ: 25 years of image analysis. Nat. Methods 9:671–675. https://doi.org/10.1038/nmeth.2089 22930834 PMC5554542

[CIT0051] Shepherd G, Stagnari F, Pisante M, et al 2008. Visual Soil Assessment – Field guide. Food and Agriculture Organization of the United Nations. ISBN: 978-92-5-105937-1. https://www.fao.org/4/i0007e/i0007e00.pdf

[CIT0052] Simonetto A, Sperandio G, Battisti A, et al 2022. Exploring the main factors influencing habitat preference of *Popillia japonica* in an area of recent introduction. Ecol. Inf. 70:101749. https://doi.org/10.1016/j.ecoinf.2022.101749

[CIT0053] Szendrei Z, Isaacs R. 2005. Do plant cues influence the oviposition behaviour of Japanese beetles? Entomol. Exp. Appl. 117:165–174. https://doi.org/10.1111/j.1570-7458.2005.00346.x

[CIT0054] Tartanus M, Malusà E, Łabanowska B, et al 2017. Utilization of non-chemical (mechanical and physical) methods to control soil-borne pests in organic strawberry plantations. J. Res. Appl. Agric. Eng. 62:182–185.

[CIT0055] Torrini G, Paoli F, Mazza G, et al 2020. Evaluation of indigenous entomopathogenic nematodes as potential biocontrol agents against *Popillia japonica* (Coleoptera: Scarabaeidae) in northern Italy. Insects 11:804. https://doi.org/10.3390/insects11110804 33202584 PMC7697182

[CIT0056] Valladares G, Lawton JH. 1991. Host-plant selection in the holly leaf-miner: does mother know best? J. Anim. Ecol. 60:227–240. https://doi.org/10.2307/5456

[CIT0058] Wickham H. 2024. ggplot2. Wiley Interdiscip. Rev. Comput. Stat. 3:180–185. https://doi.org/10.1002/wics.147

[CIT0059] Wood TN, Richardson M, Potter DA, et al 2009. Ovipositional preferences of the Japanese beetle (Coleoptera: Scarabaeidae) among warm- and cool-season turfgrass species. J. Econ. Entomol. 102:2192–2197. https://doi.org/10.1603/029.102.0623 20069848

