# Supplementary material for: Effectiveness of mulches in preventing Popillia japonica (Coleoptera: Scarabaeidae) oviposition in nursery potted plants
Source: J Econ Entomol. 2025 Jun 13;118(4):1589–98. doi: 10.1093/jee/toaf106 (PMC12449163; doi:10.1093/jee/toaf106)
Supplement: toaf106_suppl_Supplementary_Material [file toaf106_suppl_supplementary_material.docx]

**Supplementary materials**

**Supp. Table S1** Summary of data collected in the choice test.

| **Mulching materials** | **No. of pots** | **Total *P. japonica*** | **Mean *P. japonica*** | **SE** |
| --- | --- | --- | --- | --- |
| Biodegradable liquid mulch | 8 | 50 | 6.25 | 3.75 |
| L*olium* spp. | 4 | 24 | 6.00 | 2.80 |
| Vermiculite | 8 | 45 | 5.63 | 2.31 |
| Untreated | 8 | 39 | 4.88 | 2.06 |
| Pine bark | 8 | 33 | 4.13 | 1.48 |
| Rice husk | 8 | 26 | 3.25 | 1.24 |
| Beech chips | 8 | 21 | 2.63 | 1.34 |
| Perlite | 8 | 20 | 2.50 | 1.12 |
| Lapilli pebbles | 8 | 15 | 1.88 | 0.97 |
| Akadama | 8 | 13 | 1.63 | 1.07 |
| Miscanthus chips | 8 | 13 | 1.63 | 1.16 |
| Hemp chips | 8 | 10 | 1.25 | 1.11 |
| Gravel pebbles | 8 | 10 | 1.25 | 1.11 |
| Pine wood chips | 8 | 5 | 0.63 | 0.63 |
| Coconut fibre | 8 | 3 | 0.38 | 0.26 |

**Supp. Table S2** *Popillia japonica* egg-laying among different mulching materials in the choice test, expressed as the estimated mean eggs and larvae ± standard error and the 95% confidence intervals (CI 95%). Different letters indicate significant differences (*P* < 0.05) between mulching materials in pairwise comparison (without *P*-value adjustment) from the negative binomial GLM.

| **Mulching material** | **Response** | **CI 95%** | |
| --- | --- | --- | --- |
|  |  | **Lower limit** | **Upper Limit** |
| Untreated | 9.70 ± 5.6e | 3.12 | 30.30 |
| Pine bark | 6.20 ± 3.6de | 1.96 | 19.60 |
| Vermiculite | 6.20 ± 3.6de | 1.94 | 19.50 |
| Rice husk | 5.10 ± 3.0cde | 1.59 | 16.20 |
| *Lolium* spp. | 4.00 ± 3.4bcde | 0.76 | 21.20 |
| Biodegradable liquid mulch | 2.90 ± 1.8bcde | 0.86 | 9.50 |
| Perlite | 2.80 ± 1.7bcde | 0.85 | 9.40 |
| Lapilli pebbles | 1.50 ± 1.0abcd | 0.41 | 5.30 |
| Beech chips | 1.30 ± 0.9abcd | 0.36 | 4.80 |
| Miscanthus chips | 0.90 ± 0.6abc | 0.22 | 3.40 |
| Akadama | 0.90 ± 0.2abc | 0.22 | 3.40 |
| Hemp chips | 0.70 ± 0.5ab | 0.16 | 2.80 |
| Gravel pebbles | 0.70 ± 0.5ab | 0.16 | 2.80 |
| Pine wood chips | 0.40 ± 0.3a | 0.07 | 1.80 |
| Coconut fibre | 0.20 ± 0.2a | 0.04 | 1.40 |

Different letters indicate significant differences (*P* < 0.05) between mulching materials in pairwise comparison (without *P*-value adjustment) from the negative binomial GLM.

**Supp. Table S3** Summary of data collected in the no-choice test.

| **Mulching materials** | **No. of pots** | **Total *P. japonica*** | **Mean *P. japonica*** | **SE** |
| --- | --- | --- | --- | --- |
| Untreated | 4 | 120 | 30.00 | 7.80 |
| Pine bark | 4 | 318 | 79.50 | 30.80 |
| Rice husk | 4 | 285 | 71.25 | 10.40 |
| Miscanthus chips | 4 | 264 | 66.00 | 8.70 |
| Hemp chips | 4 | 254 | 63.50 | 16.00 |
| Beech chips | 4 | 213 | 53.25 | 11.10 |
| *Lolium* spp. | 8 | 342 | 42.75 | 6.20 |
| Akadama | 4 | 118 | 29.50 | 14.30 |
| Perlite | 4 | 97 | 24.25 | 4.20 |
| Vermiculite | 4 | 89 | 22.25 | 9.60 |
| Lapilli pebbles | 4 | 27 | 6.75 | 4.80 |
| Gravel pebbles | 4 | 6 | 1.50 | 1.50 |

**Supp. Table S4** The effectiveness of mulching materials in preventing *Popillia japonica* egg-laying in the no-choice test, expressed as the estimated mean eggs and larvae ± standard error and the 95% confidence intervals (CI 95 %).

| **Mulching material** | **Response** | **CI 95%** | |
| --- | --- | --- | --- |
|  |  | **Lower limit** | **Upper Limit** |
| Untreated | 30.00 ± 9.5cde | 16.08 | 55.96 |
| Pine bark | 79.50 ± 24.6f | 43.32 | 145.90 |
| Rice husk | 71.25 ± 22.1ef | 38.78 | 130.91 |
| Miscanthus chips | 66.00 ± 20.5def | 35.89 | 121.37 |
| Hemp chips | 63.50 ± 19.8def | 34.51 | 116.83 |
| Beech chips | 53.25 ± 16.6cdef | 28.87 | 98.21 |
| *Lolium* spp. | 42.75 ± 9.5cdef | 27.66 | 66.07 |
| Akadama | 29.50 ± 9.4cd | 15.81 | 55.05 |
| Perlite | 24.25 ± 7.8c | 12.92 | 45.51 |
| Vermiculite | 22.25 ± 7.2c | 11.82 | 41.87 |
| Lapilli pebbles | 6.75 ± 2.4b | 3.33 | 13.68 |
| Gravel pebbles | 1.50 ± 0.8a | 0.55 | 4.07 |

Different letters indicate significant differences (*P* < 0.05) between mulching materials in pairwise comparison (without P-value adjustment) from the negative binomial GLM.


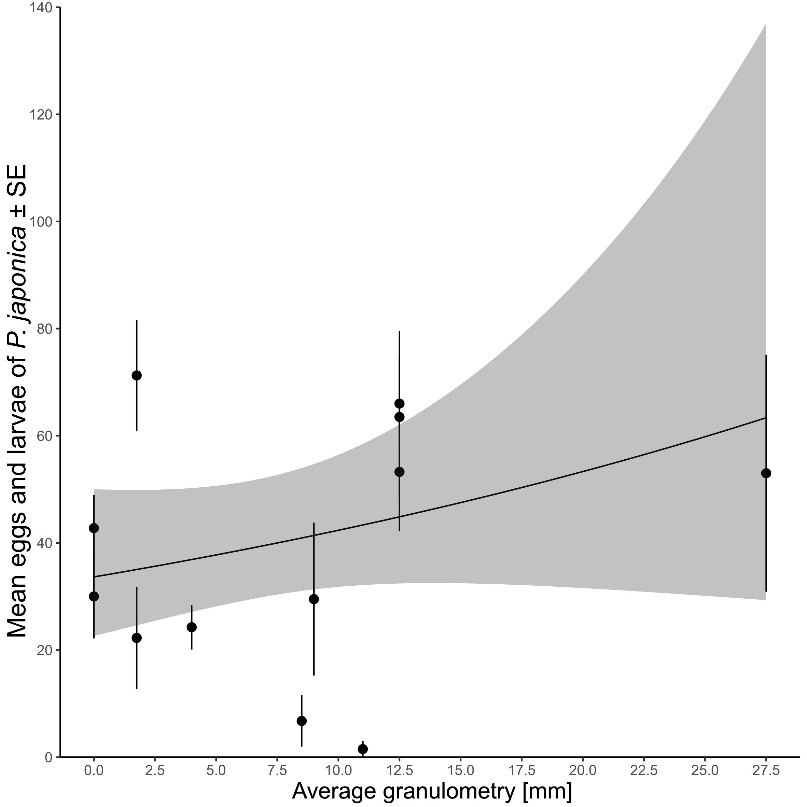


**Supp. Fig. S1** Relationship between average mulch granulometry and *Popillia japonica* egg-laying in the no-choice test (*P* = 0.22). The dots and error bars represent the average total of *P. japonica* eggs and larvae, with the standard error around the mean. The solid line represents the regression curve estimated by the model, while the shaded area indicates the 95% confidence intervals of the regression model.


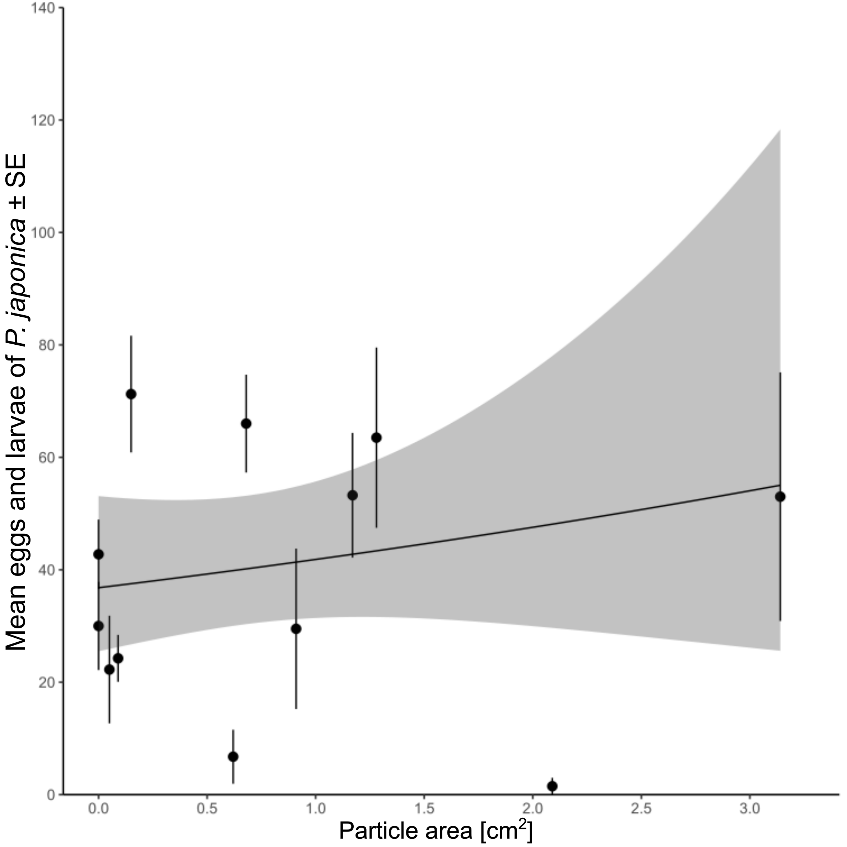


**Supp. Fig. S2** Relationship between mulch particle area and *Popillia japonica* egg-laying in the no-choice test (*P* = 0.41). The dots and error bars represent the average total *P. japonica* eggs and larvae, with the standard error around the mean. The solid line represents the regression curve estimated by the model, while the shaded area indicates the 95% confidence intervals of the regression model.

**Supp. Table S5** Number of pots exhibiting dry, medium moisture and wet soil for each mulching treatment in the choice test.

| **Mulching material** | **Soil moisture** | | |
| --- | --- | --- | --- |
|  | **Dry** | **Medium** | **Wet** |
| Untreated | 4 | 3 | 1 |
| Biodegradable liquid mulch | 4 | 3 | 1 |
| *Lolium* spp. | 3 | 1 | 0 |
| Verimculite | 1 | 5 | 2 |
| Pine bark | 1 | 4 | 3 |
| Rice husk | 3 | 4 | 1 |
| Beech chips | 4 | 3 | 1 |
| Perlite | 3 | 3 | 2 |
| Laplli pebbles | 1 | 6 | 1 |
| Akadama | 6 | 2 | 0 |
| Miscanthus chips | 2 | 3 | 3 |
| Hemp chips | 6 | 2 | 0 |
| Gravel pebbles | 6 | 0 | 2 |
| Pine wood chips | 8 | 0 | 0 |
| Coconut fibre | 5 | 2 | 1 |

The soil moisture was estimated using a visual soil assessment method, adapted from Shepherd et al. (2008). A small patch of soil was rolled by hand into a cylindrical shape (a “worm”) to assess moisture content in each pot, classifying it as follows: *Wet,* if the soil could be easily rolled into a continuous worm; *Medium,* if the worm formed but broke into several sections; and *Dry,* if the soil could not be shaped into a worm.
